# Prevalence of Obstructive Sleep Apnea Among Lebanese Patients With Chronic Kidney Disease: Its Repercussion on Disease Trajectory and Its Effect on Patients' Quality of Life

**DOI:** 10.1155/ijne/1427467

**Published:** 2025-02-23

**Authors:** Salim Yakdan, Nazih Rahhal, Soltan Al Chaar, Juliano Alhaddad, Monifa Al Akoum, Yaacoub Chahine, Robert Najem, Mirna N. Chahine

**Affiliations:** ^1^Department of Research, Faculty of Medical Sciences, Lebanese University, Hadath, Lebanon; ^2^Internal Medicine Department, Nephrology Division, Faculty of Medical Sciences, Lebanese University, Hadath, Lebanon; ^3^Nephrology Department, The Lebanese Hospital Geitaoui-UMC, Beirut, Lebanon; ^4^Basic Sciences Department, Faculty of Medical Sciences, Lebanese University, Hadath, Lebanon; ^5^Department of Research, Foundation-Medical Research Institutes (F-MRI®), Beirut, Lebanon; ^6^Department of Research, Foundation-Medical Research Institutes (F-MRI®), Geneva, Switzerland

**Keywords:** chronic kidney disease, comorbidities, Lebanon, quality of life, sleep apnea

## Abstract

**Background and Objectives:** Chronic kidney disease (CKD) remains a public health threat and a major cause of morbidity and mortality worldwide. A bidirectional relationship is found between sleep disorders and CKD worldwide. However, to our knowledge, this study is the first to assess the prevalence of obstructive sleep apnea (OSA) and to evaluate its impact on the progression of other comorbidities among Lebanese patients with CKD.

**Materials and Methods:** The study is an observational cross-sectional study, carried out between September and November 2021. Lebanese patients with any stage of CKD were included. Patients' characteristics were collected via electronic health record and baseline questionnaires. We screened for obstructive sleep apnea using the STOP-Bang questionnaire.

**Results:** We included 168 patients. The prevalence of OSA among our patients was 47.6%. The prevalence of OSA is higher in males compared with females (81.2% vs. 18.8%, *p*=0.002). Obesity was more prevalent in patients with OSA compared with patients without OSA (42.5% vs. 19.3%, *p*=0.002). Among the 168 patients, 69.6% had hypertension, with a significantly higher prevalence among those with OSA compared with those without OSA (81.2% vs. 59.1%, *p*=0.003). Patients with OSA reported significantly lower scores compared with those without OSA in several domains of physical and emotional health, including physical functioning (54.06 vs. 66.88, *p*=0.002), role limitations due to physical health (42.19 vs. 63.07, *p*=0.001), role limitations due to emotional problems (49.17 vs. 69.32, *p*=0.004), pain (61.31 vs. 70.45, *p*=0.019), and physical component score (52.53 vs. 69.53, *p*=0.002). All the abovementioned parameters were also examined in two subpopulations: patients with CKD and ESRD. Similarly, some comorbidities and a lower physical QOL score were observed more in patients with OSA in these two subpopulations.

**Conclusion:** Patients with OSA in our study have higher probability of being male, obese, and hypertensive as well as poorer QOL compared with their counterparts without OSA. Implementing more effective screening and treatment of OSA in CKD patients is necessary.

## 1. Introduction

Chronic kidney disease (CKD) remains a public health threat and a major cause of morbidity and mortality worldwide, with a global prevalence as high as 13.5% [1]. Indeed, the decline in kidney function has major repercussions on almost every organ system. The renal-sleep axis is emerging as the novel scope of recent studies [2]. Obstructive sleep apnea (OSA) is defined as complete or partial collapse episodes of the airways, associated with decreased oxygen saturation or sleep arousal. This obstruction leads to fragmented sleep, loud snoring, and witnessed apnea episodes during sleep. OSA may significantly affect cardiovascular health, mental health, and quality of life (QOL) [3–5].

Taken together, sleep disorders appear to significantly impact the prevalence of CKD [6], and this relationship is postulated to be reciprocal. First, CKD can cause OSA by narrowing the pharyngeal diameter due to fluid overload, altering chemoreflex responsiveness, and accumulating uremic metabolites [7], which can affect the tonicity of the pharyngolaryngeal muscles [8]. In addition, OSA can lead to CKD or accelerate kidney damage by causing intermittent renal hypoxia, hypertension, and sympathetic nervous system activation [7].

Furthermore, both CKD and sleep apnea have been shown to affect the renin–angiotensin system, resulting in a sympathetic overdrive and subsequent hypertension and cardiovascular morbidity [9]. Until now, despite established correlations between CKD and OSA, there are no studies investigating this relationship in the Lebanese population and especially the effect of OSA on the progression of other comorbidities and QOL in these patients. Therefore, our study aims to explore the relationship between OSA and CKD by assessing the prevalence of sleep apnea in Lebanese CKD patients, identifying comorbidities associated with OSA among this population, and evaluating the effect of sleep apnea on CKD patients' QOL.

## 2. Methods

### 2.1. Ethical Considerations

Informed consent was obtained from the participants. The nature of the data to be collected and the way these data might be used were both explained. The protection of the privacy of research participants was ensured. The Institutional Review Board (IRB) of Lebanese Hospital Geitaoui approved on our study (2021-IRB-019).

### 2.2. Study Population

The study is an observational cross-sectional study, carried out between September and November 2021. Patients were asked to fill out a questionnaire via face-to-face interviews or phone calls, and patients comorbidities and baseline characteristics were collected through electronic health records. We included adult Lebanese patients, suffering from any stage of CKD, who consented to participate in the study. Patients younger than 18 years old or those with a history of kidney transplantation were excluded. The minimal needed sample size was calculated using Cochran's formula: *n*_0_=(*Z*2 × *p* × *q*)/(*e*2 ), where *n*_0_ is the sample size, *Z* value is 1.96, *e* value is 0.05 for a 95% confidence level, and represented the prevalence of CKD in Lebanese population which is [10]. According to this formula, we needed to include a minimum of 168 patients with CKD.

### 2.3. CKD Diagnosis

Patients presenting with markers of kidney damage such as albuminuria, urine sediment abnormalities, electrolyte disturbances, and other abnormalities caused by tubular disorders, histological abnormalities, structural abnormalities detected by imaging or patients with a glomerular filtration rate (GFR) less than 60 mL/min/1.73 m^2^ (milliliters per minute per 1.73 square meters) on at least 2 times separated by at least ninety days were considered to be CKD patients [11]. Modification of Diet in Renal Disease (MDRD) Study Group equation was used to assess GFR [12, 13]. Patients with CKD were subsequently categorized into ESRD and non-ESRD groups (referred simply as CKD throughout the paper) based on their glomerular filtration rate (GFR). End-stage renal disease (ESRD) was defined as a GFR of less than 15 mL/min/1.73 m^2^ or the need for renal replacement therapy, such as hemodialysis.

### 2.4. OSA Screening

The screening for OSA was performed by using the Snoring, Tiredness, Observed apnea, high BP, BMI, age, neck circumference, and male gender (STOP-Bang) assessment tool [14].

The permission to use this assessment tool was obtained. This instrument was used to determine the prevalence of OSA among CKD patients. It consists of 8 dichotomous (yes or no) items describing the clinical features of OSA [14]. The total score is ranging from 0 to 8 with higher scores reflecting a higher risk of OSA. We chose STOP-Bang questionnaire because of its high sensitivity and specificity in identifying OSA. The sensitivity of STOP-Bang score ≥ 3 to detect moderate OSA (apnea–hypopnea index [AHI] > 15) and severe OSA (AHI > 30) is 93% and 100%, respectively, and its negative predictive values are 90% and 100%, respectively [14, 15]. Moreover, in the CKD population, a STOP-Bang score of ≥ 3 has been shown to have a sensitivity of 93% and a negative predictive value of 88% [16]. According to this questionnaire, the risk of having OSA is divided into 3 categories with [0–2]: low risk; [3–4]: intermediate risk; and [5–8] high risk. Patients with intermediate to high risk are considered as having OSA.

### 2.5. Comorbidities Definitions

Hypertension was defined as systolic BP ≥ 140 mmHg (millimeters of mercury) and/or diastolic BP ≥ 90 mmHg, measured in a sitting position or antihypertension drug use [17]. Diabetes mellitus was defined by a fasting glucose concentration ≥ 126 mg/dL (milligrams per deciliter), random glucose level ≥ 200 mg/dL, a hemoglobin A1c value ≥ 6.5%, or antidiabetic agents use [18]. Obesity was defined as a body mass index ≥ 30 kg/m^2^ (kilograms per square meter) based on the Centers for Disease Control and Prevention (CDC) definition [19]. Other comorbidities including coronary artery disease (CAD), congestive heart failure, dyslipidemia, and chronic obstructive pulmonary disease (COPD) were identified from medical records.

### 2.6. QoL Assessment

To evaluate the direct repercussion of sleep apnea in the CKD patient's QOL, the short form 36 health survey **(SF 36)** questionnaire was used [20]. It was developed at Research ANd Development Corporation (RAND). This instrument measures 8 scales: physical functioning (PF), role physical (RP), bodily pain (BP), general health (GH), vitality (VT), social functioning (SF), role emotional (RE), and mental health (MH) [20]. All items are scored so that a high score defines a more favorable health state.

### 2.7. Data Analysis

Mean and standard deviation (SD) or median and interquartile range (IQR) were reported for continuous variables and percentages were reported for categorical variables. The relationship between categorical variables was assessed with the Chi-square test. Difference in continuous variables was assessed using the *t*-test or Mann–Whitney *U* test (depending whether the variable follows a parametric or not). Variables that were significant in the univariate analysis were further analyzed using multivariate analysis. To refine the model, a model selection process was applied using the “dredge” function from the MuMIn package [21], which systematically assesses all possible models derived from the full model based on the Akaike Information Criterion (AIC). The model with the lowest AIC was selected as the best model. All statistical analyses were conducted using R Version 4.4.1. The significance level of the tests was set at a *p* value < 0.05.

## 3. Results

### 3.1. Sleep Apnea Prevalence

A total of 168 patients were included, and 80 out of 168 patients (47.6%) had a STOP-Bang score ≥ 3. The prevalence of OSA among patients with CKD was significantly lower compared with patients with ESRD (36% vs. 61.3%, *p*=0.02) (Table 1).

### 3.2. Sociodemographic and Other Characteristics of all Patients

Mean age was 61.49 years old, and there was no significant difference in age between patients with and without OSA (*p*=0.056). Our patients included 52 females (31%) and 116 males (69%). The prevalence of OSA in higher in males compared with females (81.2% vs. 18.8%, *p*=0.002).

Moreover, the mean BMI of the participants is 27.46 kg/m^2^. Patients with OSA had a significantly higher BMI than those without OSA (29.02 vs. 26.05 kg/m^2^, *p* < 0.001). Of our patients, 30.4% were obese. Obesity was more prevalent in patients with OSA compared with patients without OSA (42.5% vs. 19.3%, *p*=0.002). No difference was found in smoking status between patients with and without OSA (*p*=0.739) (Table 1).

### 3.3. Comorbidities and OSA

Among the 168 patients, 69.6% had hypertension, with a significantly higher prevalence among those with OSA compared with those without OSA (81.2% vs. 59.1%, *p*=0.003). Dyslipidemia was observed in 45.2% of the participants; however, no statistically significant difference was found between patients with and without OSA (*p*=0.181). Similarly, the prevalence of diabetes mellitus showed no significant variation between the two groups (*p*=0.796). Regarding cardiovascular diseases, CAD and congestive heart failure were reported in 45.2% and 23.2% of the patients, respectively, but neither condition differed significantly by the OSA status (*p*=0.473 and *p*=1, respectively). Finally, COPD was present in 8.9% of the patients, with no significant difference between those with and without OSA (*p*=0.462) (Table 1).

### 3.4. CKD and ESRD Subgroups

#### 3.4.1. Demographic Characteristics and Comorbidities Among Patients With CKD

In our study, patients with CKD constituted 55.35% of the total population, with 65.6% being male and 34.4% female. Among patients with CKD and with OSA, 82.4% were male and 17.6% were female, compared with 55.9% male and 44.1% female among those without OSA (*p*=0.018). Obesity was reported in 45.2% of the patients with CKD, with a significantly higher prevalence among those with OSA compared with those without OSA (76.5% vs. 27.1%, *p* < 0.001). Furthermore, patients with CKD and with OSA had a higher mean BMI than those without OSA (33.45 vs. 27.27 kg/m^2^, *p* < 0.001). However, no statistically significant differences were observed between patients with CKD and with or without OSA in terms of age (*p*=0.31), smoking status (*p*=0.856), hypertension (*p*=0.373), dyslipidemia (*p*=0.318), diabetes mellitus (*p*=0.939), CAD (*p*=0.494), congestive heart failure (*p*=0.355), or COPD (*p*=0.152) (Table 1).

#### 3.4.2. Demographic Characteristics and Comorbidities Among Patients With ESRD

Among patients with ESRD, who represented 44.65% of our cohort, 73.3% were male and 26.7% were female, with no significant gender differences between those with and without OSA (*p*=0.138). Obesity was observed in 12.0% of the patients with ESRD, but there were no significant differences in obesity prevalence or mean BMI between those with and without OSA (*p*=0.149 and *p*=0.054, respectively). Hypertension was present in 81.3% of the patients with ESRD, with a significantly higher prevalence among those with OSA compared with those without OSA (91.3% vs. 65.5%, *p*=0.013). Other factors, including smoking (*p*=0.79), dyslipidemia (*p*=0.186), diabetes mellitus (*p*=0.404), coronary artery disease (*p*=0.338), congestive heart failure (*p*=0.68), and COPD (*p*=1), did not show significant differences between patients with ESRD and with or without OSA (Table 1).

### 3.5. QOL Among all Patients

Patients with OSA reported significantly lower scores compared with those without OSA in several domains of physical and emotional health, including physical functioning (54.06 vs. 66.88, *p*=0.002), role limitations due to physical health (42.19 vs. 63.07, *p*=0.001), role limitations due to emotional problems (49.17 vs. 69.32, *p*=0.004), pain (61.31 vs. 70.45, *p*=0.019), and physical component score (52.53 vs. 69.53, *p*=0.002). However, no statistically significant differences were observed between patients with and without OSA in terms of energy/fatigue (*p*=0.448), emotional wellbeing (*p*=0.142), social functioning (*p*=0.517), general health (*p*=0.207), or mental component score (*p*=0.195) (Table 2).

### 3.6. QOL Among Patients With CKD

Patients with CKD with OSA exhibited significantly lower scores compared with those without OSA in physical functioning (58.68 vs. 69.66, *p*=0.03), role limitations due to physical health (50.74 vs. 65.68, *p*=0.041), emotional wellbeing (52.00 vs. 60.14, *p*=0.025), social functioning (50.74 vs. 59.32, *p*=0.046), and physical component score (54.17 vs. 68.45, *p*=0.047). However, no statistically significant differences were found between patients with CKD and with or without OSA in role limitations due to emotional problems (*p*=0.225), energy/fatigue (*p*=0.587), pain (*p*=0.252), general health (*p*=0.085), or mental component score (*p*=0.133) (Table 2).

### 3.7. QOL Among Patients With ESRD

Patients with ESRD and with OSA demonstrated significantly lower scores compared with those without OSA in role limitations due to physical health (35.87 vs. 57.76, *p*=0.046), pain (62.83 vs. 82.5, *p*=0.004) and physical component score (51.32 vs. 71.73, *p*=0.021). However, no statistically significant differences were observed between patients with ESRD and with or without OSA in physical functioning (*p*=0.128), role limitations due to emotional problems (*p*=0.137), energy/fatigue (*p*=0.482), emotional wellbeing (*p*=0.523), social functioning (*p*=0.758), general health (*p*=0.494), or mental component score (*p*=0.752) (Table 2).

### 3.8. Multivariate Regression Analysis

In patients with CKD, multivariate analysis identified several factors independently associated with the presence of OSA. Higher BMI was significantly associated with an increased likelihood of OSA (*β* = 0.4, *p* < 0.001). Male gender was also significantly associated with OSA (*β* = 2.2, *p*=0.002). Interestingly, lower emotional wellbeing scores was associated with the presence of OSA (*β* = −0.4, *p*=0.03) (Table 3).

In patients with ESRD, significant predictors of OSA included older age (*β* = 0.1, *p*=0.006) and higher BMI (*β* = 0.2, *p*=0.01), both of which were positively associated with OSA. Similarly, male gender was associated with an increased likelihood of OSA (*β* = 2.0, *p*=0.007). In addition, lower pain levels were inversely associated with the presence of OSA (*β* = −0.28, *p*=0.01) (Table 3).

## 4. Discussion

Our study is the first in Lebanon to estimate the prevalence of OSA and its repercussion on patients' QOL among patients with CKD. Our findings revealed that the prevalence of OSA is 47.6% among Lebanese patients with CKD. In accordance with other studies, OSA was found more prevalent in patients with ESRD compared with patients with CKD (61.3% vs. 36%) [22].

In our study, the assessment of OSA was conducted using the STOP-Bang questionnaire. While the STOP-Bang was initially validated in surgical populations and some evidence suggests that patients with ESRD may present with distinct symptoms compared to the general population [23], studies have demonstrated its utility in individuals with CKD. For instance, Nicholl et al. reported that in patients with kidney failure, a STOP-Bang score of ≥ 3 had a high sensitivity (93%) and a strong negative predictive value (88%) when using a respiratory disturbance index (RDI) threshold of ≥ 15 [16].

### 4.1. The Relationship Between Gender and Comorbidities With OSA

Male gender has been found to be more prevalent among patients with CKD and OSA. These findings are in accordance with others [24]. Studies showed that males present higher risk of CKD progression [25, 26] and OSA [27]. These differences can be justified by various reasons, mainly hormonal such as estradiol and aldosterone [28], sex differences in the pathogenesis of OSA [29], in addition to differences in upper airway anatomy and lung function between men and women [27, 30, 31].

Similarly, obesity and high BMI were found to be more prevalent among patients with CKD and OSA. These findings are in accordance with previous studies [32, 33]. Kwakernaak et al. found that abdominal fat excess was correlated with decreased GFR, decreased effective renal plasma flow, and increased filtration fraction [33]. Moreover, many studies explained that obesity presents the main risk factor for OSA and is also a risk factor for CKD. This association is due to renal function dysregulation (i.e., glomerular hyperfiltration and high albuminuria) [31, 32]. Others suggested that the inflammatory status of obese individuals affect intraglomerular hemodynamic and consequently lead to renal dysfunction [34].

In addition, our study showed that patients with CKD and OSA presented more with hypertension. These findings come in accordance with previous studies, confirming our results [35]. Studies proposed various mechanisms to justify the association between the triad: OSA, CKD, and high blood pressure. Episodes of intermittent hypoxia associated with OSA lead to the pathogenesis of hypertension [36]. Another study among patients with CKD found that patients with OSA and hypertension presents advanced CKD stage [37]. Moreover, this association can be explained by volume overload, secondary hyperaldosteronism, higher sympathetic activity, and higher inflammatory markers [38].

However, there was no statistically significant difference in terms of dyslipidemia, diabetes mellitus, CAD, and congestive heart failure. Indeed, a recent study showed that the apnea hypopnea index (AHI) is correlated with low density lipoprotein (LDL)/high density lipoprotein (HDL) ratio [38]. Previous studies explained this association by the chronic sympathetic nervous system activation caused by OSA [39, 40] that can increase the synthesis of very low-density lipoprotein (VLDL) and decreases the catabolism of LDL in the liver by stimulating *α*-1 receptors [41]. The latter lead to decreased serum HDL and increased serum LDL and, therefore, increased LDL/HDL ratio. Moreover, studies revealed that CKD progression is associated with worsening dyslipidemia [42]. A study conducted between 2001 and 2010 showed that the prevalence of dyslipidemia increases from 45.5% among CKD stage one to 67.8% among CKD stage four [43].

Finally, COPD was not statistically different between patients with CKD and with or without OSA. Although Flenley named the synergistic association between OSA and COPD as “overlap syndrome [44],” however, in accordance with our results, other studies suggested that the end-expiratory lung volume, which has an important impact on pharyngeal mechanics, and, therefore, the hyperinflation seen in COPD patients may be protective against upper airway collapse [45]. Moreover, the dilation and destruction of lung parenchyma seen in emphysema may decrease caudal traction forces, thus playing an important role in stabilizing upper airways [45].

### 4.2. The Relationship Between QOL and OSA

Overall, patients with OSA had lower QOL scores as compared with their counterparts. Physical functioning, role limitation due to physical health, role limitation due to emotional problems, emotional wellbeing, and pain had a lower scores (poorer QOL) in patients with OSA as compared with their counterparts without OSA in our population.

Our findings are similar to previous studies assessing the association between OSA and QOL. Dutt and his colleagues found that daily, social, and emotional functioning were impaired among patients with OSA [46]. D'ambrosino and his colleagues assessed QOL by SF-36 scale and found that all components of QOL (i.e., physical and mental health to social functioning) were impaired by OSA [47]. This association was explained by the number of arousals during sleep, which has been significantly correlated with mobility, cognitive and social functioning, energy, fatigue, and health distress [48].

### 4.3. Study Limitations and Perspectives

The findings of this study must be seen in light of some limitations. Our study was a cross-sectional study where the findings cannot establish a causal-effect relationship. Not all instruments employed in our study have been validated in Lebanon yet. Our findings may not be representative of the whole Lebanese population since 69% were males and were recruited from one hospital in Lebanon. In addition, individuals may have overestimated or underestimated some questions, which may result in information bias. Finally, OSA assessment was done using the STOP-Bang assessment tool, whereby we can only assess the risk of having OSA without achieving a definitive diagnosis which can only be done using polysomnographic study. Furthermore, in this assessment tool, gender, obesity, and hypertension were used to screen for OSA, so this would increase the chance for those factors to be found more in patients with OSA vs. non-OSA. Moreover, some medications such as antihypertensive drugs, benzodiazepine, opioids, hypnotics, and sedatives as well as alcohol consumption were not taken into consideration as they may have an impact on OSA and QOL of the participants.

However, several noteworthy results were underlined in this study, and it is the first, to our knowledge, in Lebanon, to highlight the effect of OSA on patients with CKD and to evaluate the QOL among Lebanese patients with CKD. The findings of this study are a step toward highlighting OSA, which is an underdiagnosed problem in Lebanese patients with CKD that can have deleterious effect on patient's comorbidities and QOL.

## 5. Conclusion

Our study was the first study conducted in Lebanon among patients diagnosed with CKD in order to assess the effect of OSA on these patients. Patients with OSA in our study have higher probability of being male, obese, and hypertensive. In addition, patients with OSA had poorer QOL compared with their counterparts without OSA. The effects of OSA observed in our study on comorbidities such as gender, weight, blood pressure, and cholesterol level among Lebanese patients with CKD should raise awareness and encourage more effective screening and treatment of OSA and related comorbidities. This, in turn, could improve patients' QOL and reduce mortality and morbidity in patients with CKD. Further studies are needed to explore the different treatment options of OSA in this population.

## Figures and Tables

**Table 1:** Sociodemographic characteristics and comorbidities of patients with and without obstructive sleep apnea (OSA).

	All patients			CKD			ESRD		
	Non-OSA	OSA	*p*	Non-OSA	OSA	*p*	Non-OSA	OSA	*p*
Number of patients (%)	88 (52.4)	80 (47.6)		59 (63.4)	34 (36.6)		29 (38.7)	46 (61.3)	
Age (mean, SD)	59.84 (11.98)	63.30 (11.21)	0.056	58.76 (11.14)	56.47 (9.01)	0.31	62.03 (13.46)	68.35 (9.98)	**0.023**
Gender (%)									
Female	37 (42.0)	15 (18.8)	**0.002**	26 (44.1)	6 (17.6)	**0.018**	11 (37.9)	9 (19.6)	0.138
Male	51 (58.0)	65 (81.2)		33 (55.9)	28 (82.4)		18 (62.1)	37 (80.4)	
Obesity (%)									
No	71 (80.7)	46 (57.5)	**0.002**	43 (72.9)	8 (23.5)	**< 0.001**	28 (96.6)	38 (82.6)	0.149
Yes	17 (19.3)	34 (42.5)		16 (27.1)	26 (76.5)		1 (3.4)	8 (17.4)	
Body mass index (mean, SD)	26.05 (4.36)	29.02 (6.06)	**< 0.001**	27.27 (4.22)	33.45 (3.85)	**< 0.001**	23.57 (3.56)	25.75 (5.28)	0.054
Smoking status (%)									
Never smoked	44 (50.0)	36 (45.0)	0.739	31 (52.5)	19 (55.9)	0.856	13 (44.8)	17 (37.0)	0.79
Former smoker	25 (28.4)	23 (28.7)		17 (28.8)	8 (23.5)		8 (27.6)	15 (32.6)	
Current smoker	19 (21.6)	21 (26.2)		11 (18.6)	7 (20.6)		8 (27.6)	14 (30.4)	
Hypertension (%)									
No	36 (40.9)	15 (18.8)	**0.003**	26 (44.1)	11 (32.4)	0.373	10 (34.5)	4 (8.7)	**0.013**
Yes	52 (59.1)	65 (81.2)		33 (55.9)	23 (67.6)		19 (65.5)	42 (91.3)	
Dyslipidemia (%)									
No	53 (60.2)	39 (48.8)	0.181	32 (54.2)	14 (41.2)	0.318	21 (72.4)	25 (54.3)	0.186
Yes	35 (39.8)	41 (51.2)		27 (45.8)	20 (58.8)		8 (27.6)	21 (45.7)	
Diabetes mellitus (%)									
No	39 (44.3)	38 (47.5)	0.796	19 (32.2)	12 (35.3)	0.939	20 (69.0)	26 (56.5)	0.404
Yes	49 (55.7)	42 (52.5)		40 (67.8)	22 (64.7)		9 (31.0)	20 (43.5)	
Coronary artery disease (%)									
No	51 (58.0)	41 (51.2)	0.473	30 (50.8)	14 (41.2)	0.494	21 (72.4)	27 (58.7)	0.338
Yes	37 (42.0)	39 (48.8)		29 (49.2)	20 (58.8)		8 (27.6)	19 (41.3)	
Congestive heart failure (%)									
No	68 (77.3)	61 (76.2)	1	40 (67.8)	19 (55.9)	0.355	28 (96.6)	42 (91.3)	0.68
Yes	20 (22.7)	19 (23.8)		19 (32.2)	15 (44.1)		1 (3.4)	4 (8.7)	
Chronic obstructive lung disease (%)									
No	82 (93.2)	71 (88.8)	0.462	53 (89.8)	26 (76.5)	0.152	29 (100.0)	45 (97.8)	1
Yes	6 (6.8)	9 (11.2)		6 (10.2)	8 (23.5)		0 (0.0)	1 (2.2)	

*Note:p* < 0.05 is considered significant. Bold values indicate a statistically significant difference between patients with OSA and those without OSA.

**Table 2:** Quality of life of patients with and without obstructive sleep apnea (OSA).

	All patients			CKD			ESRD		
	Non-OSA	OSA	*p*	Non-OSA	OSA	*p*	Non-OSA	OSA	*p*
Number of patients	88	80		59	34		29	46	
Physical functioning (mean, SD)	66.88 (25.69)	54.06 (26.40)	**0.002**	69.66 (21.79)	58.68 (25.39)	**0.03**	61.21 (31.89)	50.65 (26.89)	0.128
Role limitations: physical health (mean, SD)	63.07 (37.71)	42.19 (41.26)	**0.001**	65.68 (33.12)	50.74 (33.98)	**0.041**	57.76 (45.86)	35.87 (45.23)	**0.046**
Role limitations: emotional problems (mean, SD)	69.32 (42.35)	49.17 (46.23)	**0.004**	77.97 (37.45)	67.65 (42.23)	0.225	51.72 (46.79)	35.51 (44.67)	0.137
Energy/fatigue (mean, SD)	48.58 (13.43)	47.12 (11.10)	0.448	48.14 (13.26)	46.62 (12.29)	0.587	49.48 (13.97)	47.50 (10.26)	0.482
Emotional wellbeing (mean, SD)	61.50 (17.37)	57.35 (19.06)	0.142	60.14 (16.92)	52.00 (16.09)	**0.025**	64.28 (18.23)	61.30 (20.26)	0.523
Social functioning (mean, SD)	62.50 (23.60)	60.00 (26.34)	0.517	59.32 (20.18)	50.74 (18.95)	**0.046**	68.97 (28.66)	66.85 (29.01)	0.758
Pain (mean, SD)	70.45 (24.09)	61.31 (25.94)	**0.019**	64.53 (22.73)	59.26 (18.23)	0.252	82.50 (22.57)	62.83 (30.54)	**0.004**
General health (mean, SD)	44.89 (20.33)	41.12 (17.97)	0.207	43.47 (20.45)	36.47 (15.10)	0.085	47.76 (20.12)	44.57 (19.26)	0.494
Physical component score (mean, SD)	69.53 (35.16)	52.53 (33.44)	**0.002**	68.45 (33.75)	54.17 (31.33)	**0.047**	71.73 (38.40)	51.32 (35.21)	**0.021**
Mental component score (mean, SD)	62.23 (20.98)	57.68 (24.34)	0.195	63.01 (19.63)	56.25 (22.48)	0.133	60.63 (23.79)	58.74 (25.82)	0.752

*Note:p* < 0.05 is considered significant. Bold values indicate a statistically significant difference between patients with OSA and those without OSA.

**Table 3:** Multivariate regression analysis in patients with CKD and ESRD.

	*β* coefficient	Standard error (SE)	*Z* value	*p* value
Patients with CKD				
BMI	0.4	0.07	4.6	**< 0.001**
Male gender	2.2	0.7	3	**0.002**
Emotional wellbeing	−0.4	0.2	−2	**0.03**
Patients with ESRD				
Age	0.1	0.02	2.7	**0.006**
BMI	0.2	0.07	2.4	**0.01**
Gender	2	0.7	2.6	**0.007**
Pain	−0.28	0.11	−2.5	**0.01**

*Note:p* < 0.05 is considered significant. Bold values indicate statistical significance.

## Data Availability

The data supporting the findings of this study are available from the corresponding author upon reasonable request.
